# Image Segmentation and Analysis of Flexion-Extension Radiographs of Cervical Spines

**DOI:** 10.1155/2014/976323

**Published:** 2014-10-13

**Authors:** Eniko T. Enikov, Rein Anton

**Affiliations:** ^1^Department of Aerospace and Mechanical Engineering, University of Arizona, 1130 N. Mountain Avenue, Tucson, AZ 85721-0119, USA; ^2^Department of Surgery, University of Arizona, 1501 N. Campbell Avenue, Tucson, AZ 85724-5070, USA

## Abstract

We present a new analysis tool for cervical flexion-extension radiographs based on machine vision and computerized image processing. The method is based on semiautomatic image segmentation leading to detection of common landmarks such as the spinolaminar (SL) line or contour lines of the implanted anterior cervical plates. The technique allows for visualization of the local curvature of these landmarks during flexion-extension experiments. In addition to changes in the curvature of the SL line, it has been found that the cervical plates also deform during flexion-extension examination. While extension radiographs reveal larger curvature changes in the SL line, flexion radiographs on the other hand tend to generate larger curvature changes in the implanted cervical plates. Furthermore, while some lordosis is always present in the cervical plates by design, it actually decreases during extension and increases during flexion. Possible causes of this unexpected finding are also discussed. The described analysis may lead to a more precise interpretation of flexion-extension radiographs, allowing diagnosis of spinal instability and/or pseudoarthrosis in already seemingly fused spines.

## 1. Introduction

Spinal surgeries involving fusion of one or more vertebral pairs are among the most costly surgeries in the United States associated with the treatment of back pain and degenerative spine diseases. More than 80 percent of the population at some point in their life will be affected by back pain, which results in more than $100 billion in annual back pain related health-care expenses in the US alone. Spinal fusion surgeries, initially developed for the treatment of spinal fractures, tumor surgeries, and congenital deformities, are increasingly being used in the treatment of degenerative diseases of the spine [1, 2]. A common complication of spinal fusion is the absence of fusion, that is, the formation of pseudoarthrosis (artificial joint). Failed spinal surgeries due to the failure of fusions remain a serious problem that requires a revision surgery. For the cervical spine, the annual reoperation rate ranges between 2.5% and 6.9% based on reports from different studies [3, 4].

Computed tomography and X-ray image analyses are the main diagnostic tools for determining the cause of a failed spine surgery. Computed tomography (CT) scans can produce a more direct observation of fusion; however, the presence of metallic constructs and opaque cage materials oftentimes prevents direct observation [5]. Given the higher radiation dose of CT scans, the most common follow-up examination of cervical spine surgeries is still based on flexion-extension radiographs. These exams use two main fusion criteria [6, 7]. Both methods examine the relative motion between fused vertebrae. The Simmons criterion detects rotations of the fused vertebrae using manually selected landmark points and postulates that relative rotations exceeding 2° are considered as a sign of a failed fusion. In Hutter's method, the relative motion is examined via superposition of the flexion and extension radiographs aimed at visually observing motion between the vertebrae. Despite their wide availability, flexion-extension radiographs remain difficult to interpret. In a study in 2007, seven trained radiologists and neurosurgeons independently examined 29 flexion-extension radiographs of cervical spines with spine fusions. The interobserver agreement on the presence/absence of fusion was low (*κ* = 0.17) [8]. In this paper, we present a new analysis tool for cervical flexion-extension radiographs based on machine vision and computerized image processing. The method uses automatic image segmentation leading to detection of common landmarks, such as the spinolaminar (SL) line, known as the “fingerprint of vertebral trauma” [9, 10]. The premise of this effort is that changes in the local curvature of the spinolaminar line, as well as changes in the location of the peak curvature, can be used to diagnose trauma or possibly failure of fusion. However, validation of this conjecture is beyond the scope of the present work and is subject to future investigation.

Automatic image segmentation and identification of anatomical landmarks have the advantage of eliminating the subjectivity in the image analysis. The first attempt to develop an automatic landmark detection system of cephalograms was carried out by [11] using edge detection and line-following algorithms. Subsequent improvements included use of pattern matching techniques [12, 13]. Automatic extraction of bone contours and extraction of kinematic parameters such as Cobb angle changes in spine have been demonstrated by [14]. Their method was based on edge detection followed by Hough transform to determine the slope; however, due to irregular shapes of the vertebrae, the method failed to detect the proper lines. A more complex statistical method of 3D template matching was proposed for extracting 3D vertebreal coordinates using 3D/2D registration of biplanar radiographic images [15]. The method required the creation of a database of 1020 thoracic and lumbar vertebrae, which were then rescaled and projected onto the 2D radiographs until the projections matched the edges of the actual vertebra. While promising, the accuracy of the extracted parameters is not yet known and the authors reported sensitivity to occlusions from lungs or other organs requiring smoothing of the interrupted lines. Furthermore the method is semiautomatic as the process begins by manual identification of vertebral end-plates [15]. In an effort to reduce computational demands, a more recent study presented a semiautomatic analysis of the cervical spine using Harris corner point detection algorithm [16, 17]. This report for the first time demonstrates analysis of global curvature change of the cervical spine using healthy spines without degenerative conditions. In the case of fusion surgeries on degenerated spines, the insertion of bone grafts and other stabilization hardware often degrades the contours of the vertebra making detection of sharp corners difficult. In this paper, we present an alternative segmentation technique based on the method of *K*-means clustering [18]. The method utilizes some basic scene knowledge characteristic for flexion-extension radiographs to identify the spinolaminar line formed by the high contrast of the base of the spinous and transverse processes. With simple mirroring operation, the presented method is also suitable for identification and analysis of the implanted hardware on the anterior side of the vertebral bodies, which can be used to analyze forces and moments acting on the hardware during flexion-extension motion.

## 2. Material and Methods

Deidentified flexion-extension radiographs were obtained from the treating physician of patients who underwent cervical spine fusions at the University of Arizona Medical Center.

Image segmentation based on *K*-means clustering of gray-scale intensity levels was applied to the images to classify the pixels of the image *Ω* into *K* pixel classes, *S*_*k*_ [19]:

(1) $$\begin{array}{c} \Omega =\overset{K}{\underset{k=1}{⋃}}\;S_{k}. \end{array}$$

**Figure 1:**
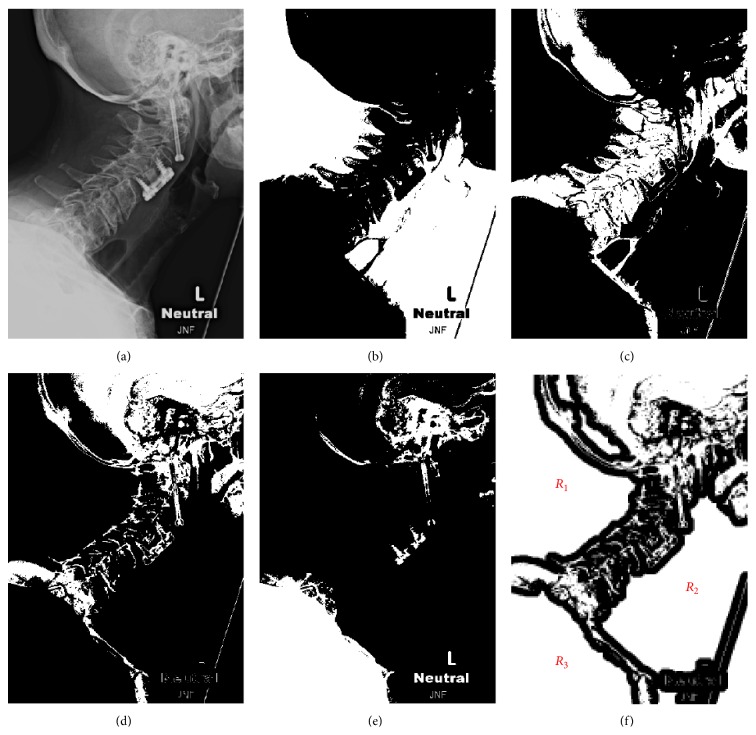
Image segmentation via *K*-means clustering: (a) original image; ((b)–(e)) *S*_*k*_, *k* = 1 ⋯ 4, respectively; (f) inverse of *S*_3_ after erosion.

The original image (see Figure 1(a)) and the resulting clusters for *K* = 4 are shown in Figures 1(b)–1(f) as black-and-white masks. It can be noticed that, despite its simplicity, this segmentation method captures the outline of the cervical vertebrae in cluster *S*_3_ (see Figure 1(d)). Furthermore, the discontinuous north-west boundary of cluster *S*_3_ captures the spinolaminar line.

The optimal number of clusters *K* and the mask *S*_*j*_ that results in the best segmentation is determined empirically. For all processed images, *K* ∈ {4,5, 6,7} and *j* ∈ {*K* − 1, *K* − 2}. In the subsequent steps, an algorithm to extract the coordinates of the spinolaminar line is described. Let the cluster of cervical vertebrae be represented by *S*_*j*_ (*j* = 3 in this example). A morphological erosion of the inverse of *S*_*j*_ is carried out using a disk structuring element *H* with diameter *d*:

(2) $$\begin{array}{c} M={\overset{-}{S}}_{j}⊖H. \end{array}$$

**Figure 2:**
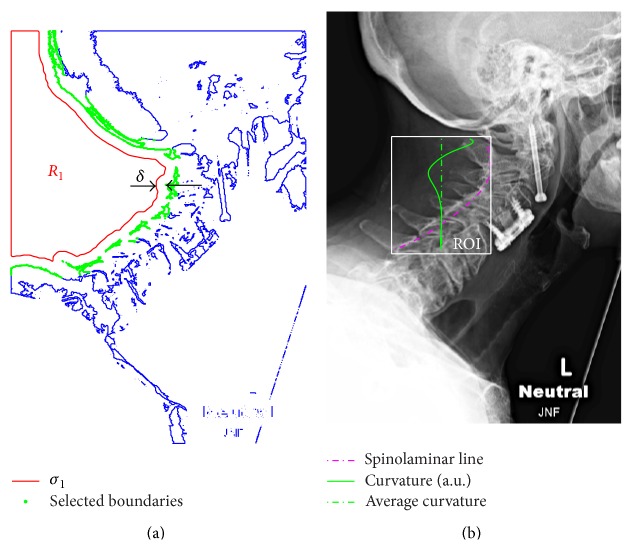
Segmentation of SL line: (a) selection of contours within distance *δ* from *σ*_1_ = ∂*R*_1_; (b) least-squares fit to SL line within ROI (dashed line) and a scaled plot of local curvature of SL (continuous trace).

**Figure 3:**
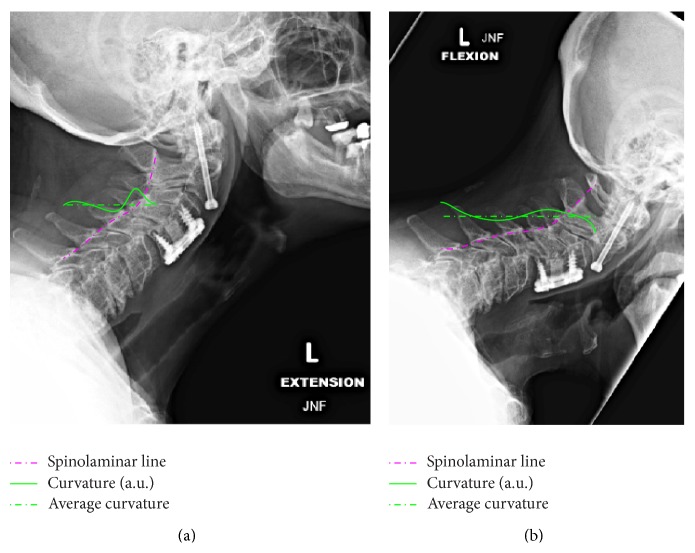
Least-squares fitted SL line (dashed line) and resulting curvature (continuous line): (a) extension; (b) flexion.

The eroded mask *M* is then subjected to a two-pass connected component labeling algorithm [20], implemented in the MATLAB bwlabel command, in order to identify the three largest regions *R*_1_, *R*_2_, and *R*_3_ (see Figure 1(f)). The boundary of the region closest to the upper left corner, *R*_1_, is then used to select the points from the original cluster *S*_*j*_. Prior to detecting the nearest points, the mask *M* is segmented into closed boundaries (shown in blue in Figure 2(a)). Among these boundaries only those lying within a distance *δ* are considered part of the spinolaminar line (see points in green in Figure 2(a)). The selected points are superimposed in red over the original image in Figure 2(b). As evident, in addition to the spinolaminar line, the selection includes a section from the skull, as well as the T1 vertebrae. Figure 2(b) also contains a curvature plot (green line), described in the next section. At this stage, the selection of the region of interest (ROI) encompassing the SL line is carried out manually (see Figure 3(a)); however, automation is possible by identifying the convex corner appearing between the skull and C1. Points from the SL line within the ROI are then used to fit a sixth-order polynomial *f*(*s*), where *s* is either an *x* or *y* coordinate. The coefficients of the obtained polynomial are subsequently used to obtain differential geometric parameters, such as local curvature and slope.

## 3. Experimental

**Figure 4:**
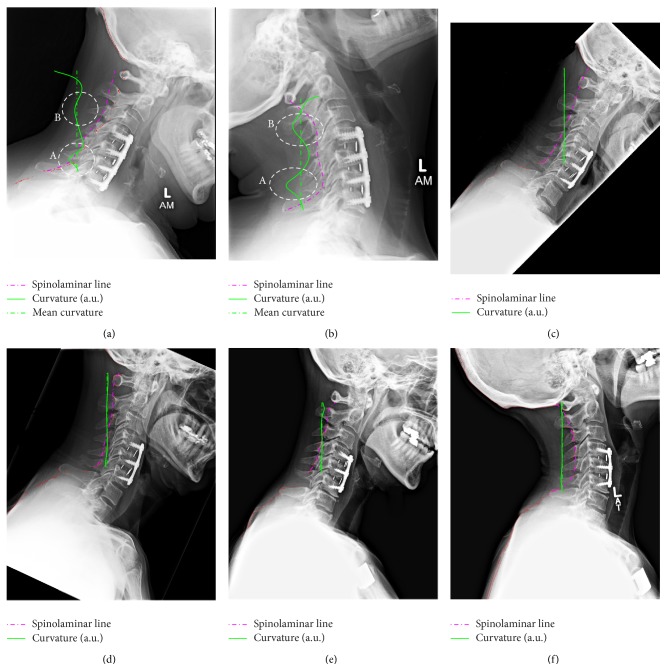
Fitted SL line (dashed line) and resulting curvature (continuous line) from a second subject: (a) flexion; (b) extension. Adjacent levels are marked with A and B, respectively.

The method described in the previous section was applied to two additional radiographs from the same subject.  Figure 3 shows the resulting SL line segmentation. The parameters *K*, *j*, *d*, and *δ* for each image are listed in Table 1. The method was tested on three additional subjects with cervical fusion surgeries over a total of 6 additional images. The resulting SL line and its curvature are shown in Figure 4, while the image segmentation parameters are listed in the last six rows of Table 1.

To analyze changes in the curvature of the selected SL line, its points are ordered in monotonically increasing order along *x* or *y* to allow curve-fitting. Any duplicates are removed. The resulting set is used to fit a sixth-order polynomial in the form

(3) $$\begin{array}{c} f\left(s\right)=a_{6}s^{6}+a_{5}s^{5}+a_{4}s^{4}+a_{3}s^{3}+a_{2}s^{2}+a_{1}s+a_{0}, \end{array}$$

where *s* represents the ordered coordinate direction. The local curvature is then computed using

(4) $$\begin{array}{c} c\left(s\right)=\frac{\ddot{f}}{{\left(1+{\dot{f}}^{2}\right)}^{3/2}}, \end{array}$$

where $\dot{f}=df/ds$. The resulting SL line fit and its curvature are overlayed over the radiographs as dotted (magenta) and continuous (green) traces, respectively (see Figures 2–4). The curvature trace has been rescaled in nondimensional units 5/*L*, where *L* is the length of the cervical plate in each image. The curvature plots show a localized increase in the local curvature at points adjacent to the cervical plate, which is consistent with the hypothesis of higher utilization of adjacent segments and associated adjacent level disease [21].

## 4. Analysis

The proposed curvature analysis technique can be useful in determining local motion abnormalities. In clinical literature, spinal curvature commonly implies changes of the interverterbal angles (Cobb angles). The local curvature of a beam, *κ*, is related to the change of angular orientation of the tangent line, *θ*, through

(5) $$\begin{array}{c} \kappa =\frac{d\theta \left(s\right)}{ds}, \end{array}$$

where *s* is the arc-length coordinate along the beam. It is apparent that, due to the additional differentiation in (5), the curvature is more sensitive to changes than the angle *θ*. It is easy to see from (5) that this product produces the total rotation between the two ends of the SL line

(6) $$\begin{array}{c} \left(s_{2}-s_{1}\right)\overset{-}{\kappa }=\overset{s_{2}}{\underset{s_{1}}{\int }}\kappa =\theta \left(s_{2}\right)-\theta \left(s_{1}\right). \end{array}$$

Therefore, the average curvature captures the total rotation between the two ends of the spinal section under study. Thus comparing differences between local and average curvature could be used as an indicator of excessive local deformation. For example, a comparison between Subject 4 (Figures 4(e)-4(f)) and Subjects 1–3 (Figures 3, 4(a)–4(d)) clearly shows a more uniform curvature distribution in the former in comparison to the latter. If one considers the spine as an elastic beam, its curvature can be related to the bending moment, *M*(*s*), and the beam's local rigidity, EI(*s*), through

(7) $$\begin{array}{c} \text{EI}\left(s\right)=\frac{M\left(s\right)}{\kappa \left(s\right)}. \end{array}$$

Therefore, local reduction of spine flexural rigidity can be related to rapid changes in the local curvature, provided that the internal bending moment *M*(*s*) does not change as rapidly. While the latter is a hypothesis to be tested in the case of human spines, using variations of local curvature, Ratcliffe was able to detect cracks in elastic beams that are 2% of its total thickness [22]. Within this limited study, it appears that Subject 4 showed a more uniform curvature in flexion and extension, implying an optimal distribution of the deformation in both cases.

**Figure 5:**
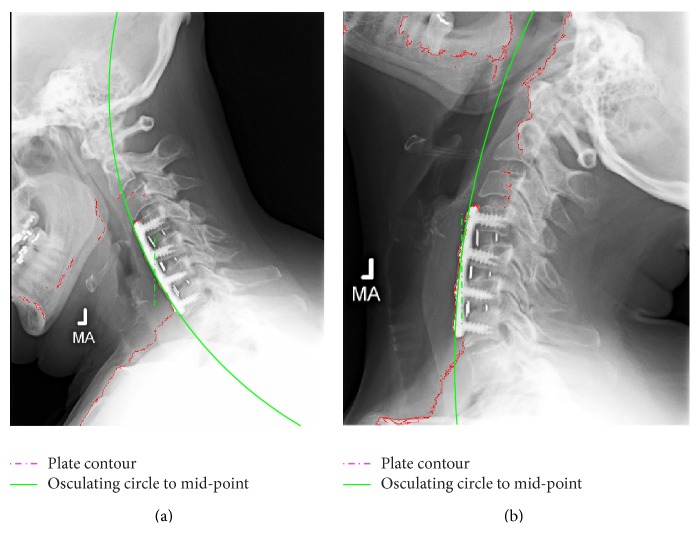
Fitted plate contour (dashed line) and osculating circle (continuous) fitted to the plates mid-point: (a) flextion; (b) extension.

**Figure 6:**
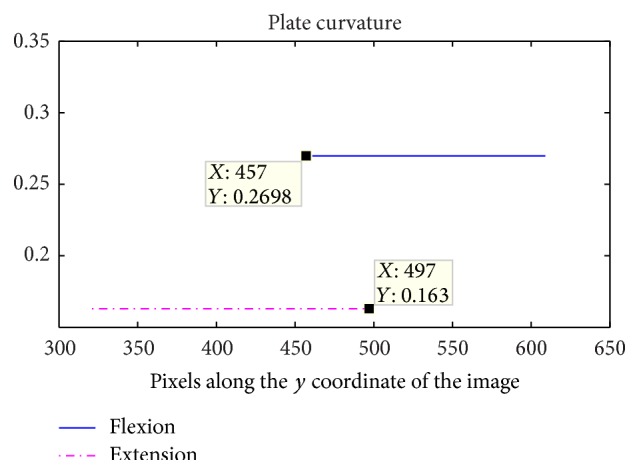
Average curvature measured in 1/*L* during flexion (solid line) and extension (dashed line).

**Figure 7:**
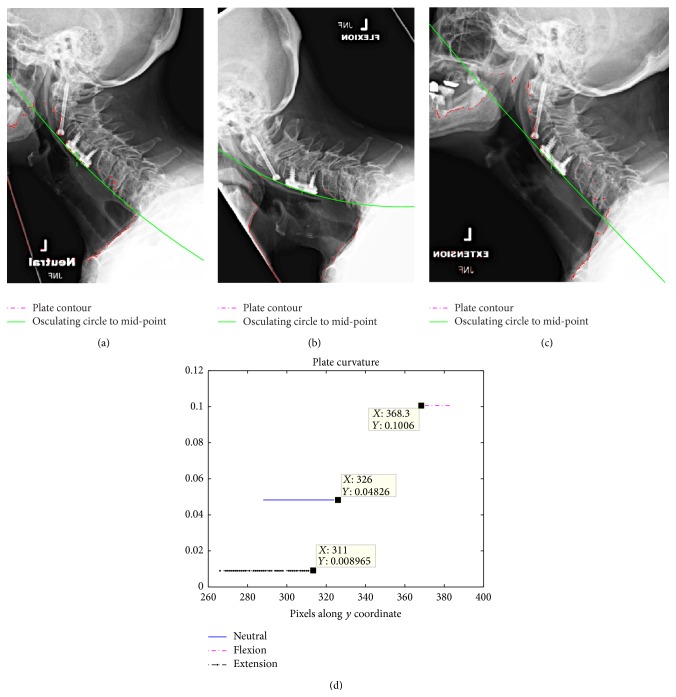
Fitted plate contour (dashed line) and osculating circle (continuous) fitted to the plates mid-point: (a) neutral; (b) flexion; (c) extension; (d) plate curvatures in 1/*L* units.

The proposed image segmentation method was also used to analyze the deformation of the cervical plates. A simple mirroring of the image prior to segmentation analysis allows the same algorithm to be applied to the anterior contour of the vertebrae including the implanted hardware.  Figure 5 shows the mirrored images after the application of the segmentation method. The continuous line (green) marks the osculating circle fitted to the mid-point of the cervical plate. In order to obtain the average curvature, the polynomial (3) was truncated to second-order terms. A comparison between the average curvature of the plate during extension and flexion is shown in Figure 6. The corresponding radii of curvature are 3.7*L* and 6.1*L* for flexion and extension, respectively. These values are within the manufacturer's built-in lordotic radii or 2–5*L*; however, it is surprising that noticeable plate curvature changes occur during flexion-extension motion. Furthermore, it is apparent that these changes occur in the opposite direction to the changes in the cervical spine curvature, that is, the curvature becomes more lordotic during flexion compared to extension. This prompted a similar analysis on the images of Subject 1 with a single-level fusion. The obtained plate curvatures from flexion, neutral, and extension radiographs are shown in Figure 7. It is apparent that the extension leads to the smallest lordotic curvature, while the flexion produces the largest.

## 5. Results

The developed semiautomatic image segmentation method allows for quantitative analysis of flexion-extension radiographs. Four tuning parameters were used to segment the images. Examples of extracted features include the SL line and cervical plate contours. These were analyzed using the methods of differential geometry in order to obtain local curvature changes. The method provides an ability to analyze changes in the curvature of the SL line and pinpoint the points of largest curvature change. Often, these occur in the levels adjacent to the fused vertebrae. Analysis of the curvature changes of the plates shows that, contrary to the expectation that the plates would follow the contour of the spine, minute curvature changes occur in the opposite direction; that is, for lordosis increasing motion, the lordosis in the plate diminishes. Possible explanations for this difference are settling of the graft during fusion, leading to the transfer of higher loads in the caudal-cephalic directions through the rigid plate-screw construct. Such higher compressive loads have been described in [23], where the authors report a 10-fold increase of compressive stresses during flexion (kyphotic configuration) in comparison to the magnitude of tensile stresses during extension (lordotic configuration). The lack of redistribution of these large compressive stresses as a result of using rigid constrained plates is the primary motivation for the development of dynamic plates allowing motion of the fixation screws [24].

Given that the rigidity of the plate-screw system is generally known, the presented image analysis technique could lead to in situ force/bending moment measurements, too. Using curvature instead of Cobb angles in the analysis of flexion-extension radiographs tends to result in an increase of the sensitivity due to the curvature being proportional to the second derivative of displacements. However, the method also amplifies errors in the segmentation and therefore should be used with caution. Segmentation errors generally occur at C7, where the extracted contour deviates from the SL line and “drifts” along the spinous process of C7 (see Figure 4). Other errors occur at the junction with the skull in extension radiographs, when the posterior arch of the atlas (C1) overlaps with the occipital bone. In such cases, the exact location of the cephalic end of the SL line is lost (see Figure 3(b)).

## 6. Conclusions

This work presented a semiautomatic image analysis of conventional flexion-extension radiographs. The presented method allows for quantitative analysis of the motion of individual vertebrae and implanted hardware. The average total time for image segmentation and processing was 3.53 seconds; however on average 3-4 runs were used to determine the optimal values of the parameters from Table 1. The increased sensitivity of the curvature-based analysis may lead to more accurate interpretation of flexion-extension radiographs and the diagnosis of pseudoarthrosis. Further applications include in situ force estimation and the development of curvature-based criteria for fusion and analysis of spinal instability. While limited in size, the present study illustrates the ability of the method to detect larger curvature changes adjacent to the fused vertebrae, which are commonly associated with adjacent level disease. Future efforts should be aimed at replacing the manual selection of the optimal segmentation parameters with automatic selection using machine learning algorithms. Further improvements of the efficiency can be obtained by selecting the ROI earlier in the image processing sequence. Last but not least, the technique should be applied to biplanar images to reduce any project errors in the estimation of the curvature.

Within this limited study, Subject 4 data showed consistently uniform curvature distribution in flexion and extension radiographs, implying an optimal distribution of the deformation in both cases, while Subjects 1–3 showed consistent variation in the local curvature of the SL line. Possible clinical relevance of this finding could imply a better distribution of the total range of motion for Subject 4 over Subjects 1–3 and lower likelihood of developing adjacent level disease; however a larger study should be undertaken to validate this hypothesis. Such studies will likely be carried in supine position in order to ensure laxity of the neck muscles and, therefore, uniform bending moments of the cervical spine.

## Figures and Tables

**Table 1:** Segmentation parameters.

Image	*K*	*j*	*d*, pixels	*δ*, pixels
Subject 1 Figure 2(b) (neutral)	4	3	13	26
Subject 1 Figure 3(b) (flexion)	6	4	14	26
Subject 1 Figure 3(a) (extension)	6	5	13	18
Subject 2 Figure 4(a) (flexion)	6	5	20	23
Subject 2 Figure 4(b) (extension)	6	5	13	26
Subject 3 Figure 4(c) (flexion)	6	4	68	82
Subject 3 Figure 4(d) (flexion)	7	4	45	69
Subject 4 Figure 4(e) (flexion)	7	4	45	69
Subject 4 Figure 4(f) (extension)	6	4	45	69
